# Emerging Roles of Long Noncoding RNAs in the Cytoplasmic Milieu

**DOI:** 10.3390/ncrna6040044

**Published:** 2020-11-09

**Authors:** Michelle Aillaud, Leon N Schulte

**Affiliations:** 1 Institute for Lung Research, Philipps University Marburg, 35043 Marburg, Germany; Aillaud@students.uni-marburg.de; 2 German Center for Lung Research (DZL), 35392 Giessen, Germany

**Keywords:** Long noncoding RNA, lncRNA, cytoplasm, cytosol, translation, organelles, phase-separation

## Abstract

While the important functions of long noncoding RNAs (lncRNAs) in nuclear organization are well documented, their orchestrating and architectural roles in the cytoplasmic environment have long been underestimated. However, recently developed fractionation and proximity labelling approaches have shown that a considerable proportion of cellular lncRNAs is exported into the cytoplasm and associates nonrandomly with proteins in the cytosol and organelles. The functions of these lncRNAs range from the control of translation and mitochondrial metabolism to the anchoring of cellular components on the cytoskeleton and regulation of protein degradation at the proteasome. In the present review, we provide an overview of the functions of lncRNAs in cytoplasmic structures and machineries und discuss their emerging roles in the coordination of the dense intracellular milieu. It is becoming apparent that further research into the functions of these lncRNAs will lead to an improved understanding of the spatiotemporal organization of cytoplasmic processes during homeostasis and disease.

## 1. Introduction

The RNA pool of mammalian cells primarily consists of ribosomal RNA, followed by other types of RNA, such as messenger RNA (mRNA), transfer RNA (tRNA), small nuclear RNA (snRNA) or microRNA (miRNA). A common feature of these RNA classes is their association with distinct cellular machineries and RNA maturation pathways [1]. Long noncoding RNAs (LncRNAs), which occupy up to ~0.2% of the cellular RNA pool and are loosely defined as noncoding RNAs (ncRNAs) ≥ 200 nucleotides (nts) [1], have only recently appeared on the scene as a large class of RNA with critical implications in a multitude of cellular processes and diseases [2,3,4].

Individual ncRNAs exceeding the 200 nt length threshold were already discovered and characterized as early as in the 1980s and recognized as critical components of diverse cellular machineries. Examples are the signal recognition particle (SRP) RNA, which serves as a scaffold for SRP assembly [5], the RMRP lncRNA, which is a component of mitochondrial RNA-processing endoribonuclease MRP [6], or the starvation-induced lncRNA Gas5 [7]. Several other ncRNAs ≥ 200 nts were already discovered and characterized before the systematic annotation of lncRNA genes [7]. The recent high-throughput sequencing-based cataloguing of mammalian transcriptomes by consortia, such as ENCODE or FANTOM has additionally revealed thousands of lncRNAs [8,9], which had been overlooked in the past, possibly due to their lower median expression and higher tissue specificity compared to mRNAs [8,10]. Many of these lncRNAs have already been implicated in key cellular processes such as X chromosome silencing, differentiation or antipathogen defense through manifold engagements with protein complexes [2,11,12]. Subcellular localization of lncRNAs is tightly regulated and may depend on nuclear retention motifs [13]. Unlike early studies suggested, lncRNAs do not seem to primarily function in the nucleus but also locate to the cytoplasm in large quantities [14,15,16]. Irrespective of their subcellular localization, lncRNAs were shown to interact with proteins or RNA to function e.g., as scaffolds for ribonucleoprotein complex (RNP) assembly or decoys preventing proteins or RNAs from interaction with other molecules [11]. Furthermore, lncRNAs can guide their interaction partners to RNA or DNA to promote or repress transcription, RNA stability or translation [11] (Section 2 and Section 8). In some cases, ncRNAs, such as upstream antisense RNAs do not seem to have a regulatory function that goes beyond the act of their transcription [17]. However, the many documented functional interactions of lncRNAs with other biomolecules and their nonrandom subcellular localization suggest that the latter case does not apply to the majority of lncRNAs.

**Figure 1:**
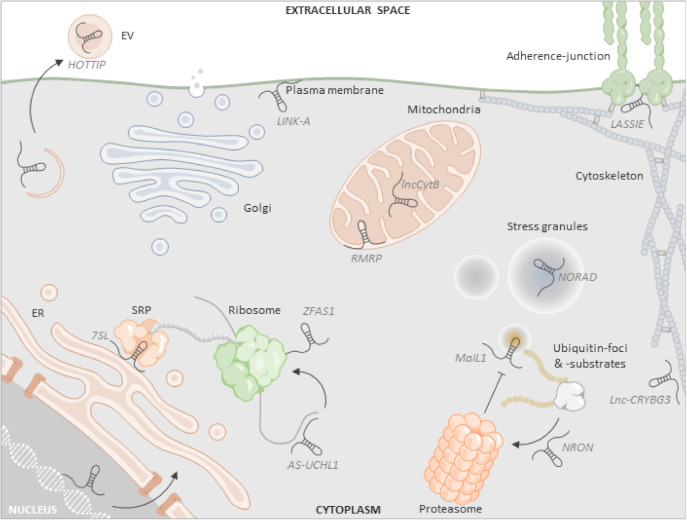
Exemplary illustration of lncRNAs in cytoplasmic structures and machineries. Many lncRNAs are exported from the nucleus and subsequently shuttled to specific cytoplasmic locations. Representative examples of lncRNAs are shown. Ribosome assembly and translation, for instance, is regulated by lncRNAs such as ZFAS1 or AS-UCHL1. At the endoplasmic reticulum (ER), the 7SL RNA scaffolds the signal recognition particle (SRP) to promote co-translational protein translocation. Several lncRNAs, including RMRP and lncCytB regulate mitochondrial translation and energy balance. LncRNA HOTTIP is packaged into extracellular vesicles (EVs) and was suggested as a liquid biopsy marker in colorectal cancer. LINK-A, LASSIE and lnc-CRYBG3 associate with the plasma membrane, adherence junctions and the cytoskeleton, respectively, as regulatory or architectural elements. Formation of cytosolic aggregates, including stress granules and ubiquitin foci, involves lncRNAs such as NORAD or MaIL1. Ubiquitin-dependent proteasomal degradation is regulated by several lncRNAs, including NRON. Systematic proximity labelling studies suggest further lncRNAs to be shuttled to distinct cytoplasmic destinations, including the ER and the Golgi apparatus, but remain to be functionally characterized.

The relatively loose definition and variety of different molecular functions raises the question of whether subgroups of lncRNAs, which can be mechanistically assigned to specific subcellular machineries or organelles, should be separated from the generic term “lncRNAs” and given their own function-related designations. In support of this view, multiple lncRNA subgroups with characteristic sedimentation rates can be discriminated on density gradients [16]. At present, the functions of most of the lncRNAs discovered in the course of the systematic ENCODE and FANTOM annotations are not yet sufficiently understood to be able to derive definable subclasses. Through individual biochemical characterization of lncRNAs, however, recurrent regulatory and architectural principles and their involvement in different cellular machineries and organelles in the nucleus and in the cytoplasm have been revealed. Recently, a variety of tools and databases have been introduced (Table 1), which are expected to facilitate the allocation of lncRNA functions to specific subcellular compartments and machineries. Examples are proximity labelling methods, such as ascorbate peroxidase (APEX)-seq or CRISPR-assisted RNA–protein interaction detection (CARPID), where a biotin-ligase labels the desired subcellular structure through gene-fusion or Clustered Regularly Interspaced Short Palindromic Repeats (CRISPR)-assisted recruitment for subsequent streptavidin-based purification and protein and RNA analysis [18,19]. Furthermore, centrifugation-based techniques such as Grad-seq (gradient sequencing), CeFraSeq (cell fractionation sequencing) and R-DeeP (RNA-dependent proteins) exist, charting the global RNA-protein co-sedimentation landscape, the shuttling of RNA to subcellular fractions and the RNA-dependence of protein complexes, respectively [16,20,21,22,23]. Such approaches were recently summarized elsewhere [24,25] and are not reiterated in detail in the present review. Also, common molecular principles guiding lncRNA interaction with individual molecules (proteins, mRNAs and microRNAs) in the cytoplasm and nucleus have recently been reviewed [2,11,12]. The present review focuses on the emerging architectural and coordinating functions of lncRNAs in cytoplasmic structures and machineries (Figure 1) and discusses possible lessons for our understanding of subcellular organization.

## 2. Coding Potential and Ribosome Association of Cytoplasmic lncRNAs

Recent studies have questioned the classic narrative that lncRNAs mostly act in the nucleus. Using subcellular fractionation techniques, a large percentage of lncRNAs was found to be exported to the cytoplasm and, at least in some cell types, the number of predominantly cytoplasmic lncRNAs seems to outnumber the nuclear-enriched lncRNAs [14,15,16]. This raises the question of the ribosome association and coding status of these lncRNAs. Through density gradient and ribosomal pull-down approaches, independent studies have estimated ~20–40% of the cellular lncRNAs to interact with ribosomal mono- or polysomes [14,15,16,32]. Of note, ribosome-association alone does not necessarily indicate productive translation of lncRNAs. Using ribosome profiling, which determines the ribosome occupation of cellular transcripts at single nucleotide resolution [33], Guttman et al. therefore re-examined the coding status of lncRNAs by making use of the observation that a stop-codon at the end of an open reading frame (ORF) is typically followed by a sharp drop in ribosome occupancy. According to the authors, this signature accurately discriminates messengers from noncoding RNAs, including lncRNAs. The authors furthermore reasoned that the ribosome occupancy observed for several lncRNAs likely reflects nonproductive initiation of translation or ribosomal scanning without translation [34]. Follow-up studies, however, suggest that a considerable number of lncRNAs may encode previously unrecognized microproteins, several of which accumulate at distinct subcellular structures, including mitochondria, the ER or the plasma membrane, reproducibly interact with other proteins and are functional [32,35]. Whether the presence of functional micro-ORFs in individual lncRNAs excludes their simultaneous regulatory participation in cellular processes independent of the translation machinery remains to be determined.

Besides random ribosomal scanning events or active translation of micro-ORFs, the association of lncRNAs with ribosomes may also be attributed to regulatory interactions, serving the adjustment of mRNA translation. LincRNA-p21 for instance, was reported to associate with JUNB and CTNNB1 mRNAs to lower their translation through recruitment of the translational repressor RCK [36]. Similarly, AdipoQ AS lncRNA was reported to bind to AdipoQ mRNA in the cytosol to suppress its translation, thereby inhibiting adipogenesis [37]. PU.1 AS lncRNA was found to promote adipogenesis by binding to the messenger of the PU.1 transcription factor, thereby inhibiting its translation [38]. Other lncRNAs promote translation through association with mRNAs, mono- and polysomes. Antisense Uchl1 RNA is an lncRNA shuttled from the nucleus to the cytoplasm in response to cellular stress and promotes polysome association and thus translation of Uchl1 mRNA [39]. LncRNA GAS5, which is present in both the nucleus and the cytoplasm [40], was reported to interact with initiation factor eIF4E and to promote c-Myc mRNA association with polysomes [41]. Cytoplasmic lncRNA ZFAS1 was found to interact with the 40S ribosomal subunit, possibly regulating ribosome assembly and translation. The potential role of ZFAS1 in this process, however, demands further mechanistic investigation [42].

Taken together, besides occasional ribosomal scanning or productive translation, several cytoplasmic lncRNAs associate with active or assembling ribosomes (Figure 1) and positively or negatively regulate protein production. So far, however, relatively few examples of lncRNAs involved in translational control are described in the available literature. An important task in the coming years will therefore be to dissect the global regulatory impact of ribosome-associated lncRNAs on protein production and separate translation-controlling lncRNAs from actively translated, micro- and noncanonical ORF-containing lncRNAs.

## 3. Localization of lncRNAs to Mitochondria

Mitochondria are not only essential to cellular energy balance but also participate in cell death programs and in signal transduction in response to environmental changes. Upon detection of pathogens by intracellular innate immune receptors or during removal of damaged mitochondria for instance, proteins such as MAVS or OPTN establish signaling platforms on the mitochondrial outer membrane, which trigger TBK1 kinase dependent type I interferon immune responses and autophagy, respectively [43]. Several lncRNAs, including MaIL1 or Lnczc3h7a, act near these signaling platforms [16,44,45]. Other lncRNAs, such as HOTAIR or TUG1, regulate the expression of nuclear-encoded subunits of the oxidative phosphorylation (OXPHOS) complexes or participate in mitochondria-induced apoptosis [46]. Aside from lncRNAs acting in the periphery of mitochondria, several lncRNAs are encoded in the mitochondrial genome or are imported into mitochondria.

By systematic transcriptome profiling, three lncRNAs were found to be encoded in the mitochondrial DNA, complementary to the ND5, ND6 and CytB mRNAs. Expression of these mitochondria-retained lncRNAs, termed lncND5, lncND6 and lncCytB, depends on their processing by mitochondrial RNaseP. Resistance to the ssRNA-specific nuclease RNase I^f^ indicated that all three lncRNAs form RNA duplexes [47]. lncND5, lncND6 and lncCytB were therefore speculated to regulate expression of their complementary mRNAs [47], which, however, remains to be proven experimentally. Further lncRNAs are potentially encoded in the mitochondrial genome, including the heart-failure-associated biomarker LIPCAR, which partially overlaps with lncCytB [48,49]. However, extensive homology to nuclear-encoded sequences demands that the postulated mitochondrial origin of LIPCAR be re-examined [49]. A nuclear-encoded lncRNA with an important role in mitochondrial energy balance is RMRP. This lncRNA was characterized as an RNA component of the mitochondrial endoribonuclease MRP, which is involved in rRNA maturation and regulates oxygen consumption [6,50]. Nuclear export of RMRP is governed by RNA binding protein (RBP) HuR, while mitochondrial matrix localization of RMRP depends on the RBP GRSF1 [50]. Mutations in the RMRP locus and copy number alterations were associated with cancer [51]. Another cancer-relevant lncRNA locating to mitochondria and regulating mitochondrial function is SAMMSON. In melanoma patients, SAMMSON is recurrently cogained with the MITF oncogene during focal amplification of chromosome 3p13-3p14 [52]. Mechanistically, SAMMSON interacts with the mitochondrial metabolic regulator p32 and promotes its mitochondrial localization. p32 is required for mitochondrial 16S rRNA maturation. SAMMSON knockdown interfered with this process and led to reduced expression of mitochondria-encoded proteins. Furthermore, SAMMSON knockdown decreased OXPHOS activity and mitochondrial membrane potential and impaired melanoma cell viability [52]. During resolution of acute inflammatory responses, the nuclear-encoded lncRNA lncFAO locates to mitochondria of murine phagocytes to promote fatty acid oxidation, which in turn reduces proinflammatory gene expression. Consequently, lncFAO was suggested to contribute to resolution of inflammation [53]. Thus, mammalian mitochondrial activity levels are tuned by mitochondrial and nuclear-encoded lncRNAs (Figure 1) with relevance to cancer and inflammatory diseases. The cosedimentation of several presently uncharacterized lncRNAs with RMRP and mitochondrial protein-components on glycerol density gradients [16] suggests further, as yet undiscovered roles of lncRNAs in mitochondria and cellular energy balance.

## 4. LncRNA Association with the Secretory and Extracellular Vesicle System

While several lncRNAs are known to act in mitochondria, little is known about the potential roles of lncRNAs in other organelles, such as the endoplasmic reticulum (ER) or the Golgi apparatus. Several lncRNAs were reported to act in their periphery, e.g., regulating ER-dependent autophagy [54] or ER-stress [55,56,57]. However, these lncRNAs do not necessarily locate to the ER. A noncoding RNA ≥ 200 nts, which directly interacts with the ER surface, is the 7SL RNA, also known as SRP RNA. The signal recognition particle (SRP) is an RBP complex, mediating the cotranslational translocation of proteins destined for secretion into the ER lumen [58]. In 1982, Walter and Blobel reported the SRP to contain a ~300 nt long noncoding RNA, which serves as an essential scaffold for SRP assembly [5,58]. More recently, lncRNA SENCR was suggested to foster ER-localization of the protein CKAP4, which anchors the ER to the cytoskeleton [59]. Similarly, lncRNA LASSIE colocalizes with ER markers [60]. While the mechanisms, through which both SENCR and LASSIE control adherence-junction formation in endothelial cells are well-documented (see Section 5), their possible roles at the ER demand further investigation. Several recent studies have systematically charted ER-localized transcripts through high-throughput FISH and proximity labelling approaches. Besides the expected enrichment of mRNAs encoding secreted proteins, however, only few lncRNAs could be detected in close proximity to the ER [18,61,62]. Thus, besides 7SL and possibly SENCR and LASSIE, only few noncoding RNAs seem to adopt ER-specific subcellular localization patterns. While ncRNA-localization to transport and secretory vesicles produced by the ER and Golgi network remains to be determined, many recent studies have identified ncRNAs within extracellular vesicles (EVs). Biogenesis of EVs primarily depends on endocytosis and autophagy pathways. Although interactions of these pathways with the ER and Golgi network exist, ER and Golgi markers are generally low on EVs [63,64]. Loading of EVs generated by the autophagic and the endosomal pathway, respectively, with RBPs and noncoding RNAs seems to occur nonrandomly [65,66]. The process of autophagy itself is tightly controlled by several lncRNAs, such as Gas5 or HULC [67]. The physiological reasons for an enrichment of specific small and long noncoding RNAs in EVs remain under debate. One hypothesis is that EVs transfer lncRNAs to other cells, to regulate recipient cell functions [66]. Regardless of the reasons for their appearance in vesicles, EV-enriched lncRNAs were suggested as biomarkers with diagnostic and prognostic value, hinting at their utility in liquid biopsy approaches [68]. In the context of colorectal cancer, for instance, low abundance of lncRNA HOTTIP in small EVs (exosomes) was associated with decreased patient overall survival [69]. Similar to the association of lncRNAs with the ER and Golgi network, however, further studies are needed to clarify the roles and clinical utility of lncRNAs enriched in EVs.

## 5. LncRNA Association with the Plasma Membrane

Besides their association with vesicles and membranes of intracellular organelles, several lncRNAs were shown to interact with phospholipids or adherence-junction proteins at the plasma membrane (Figure 1). To systematically determine lipid-bound lncRNAs, Lin et al. combined a cell fractionation technique enriching the lipid bound RNA pool, and microarray hybridization [70]. Among nine lncRNAs enriched ≥ fourfold in the lipid-associated compared to the total RNA fraction this strategy identified LINK-A as a phosphatidylcholine (PC) and phosphatidylinositol-3,4,5-trisphosphate (PIP3) phospholipid binding lncRNA. LINK-A promotes the interaction of PIP3 with AKT kinase and thereby promotes activation of this protein. In line with the critical role of the PIP3-AKT pathway in cell proliferation, hyperactivation of AKT by LINK-A was shown to promote tumorigenesis and cancer cell resistance to AKT inhibitors [70]. In endothelial cells, SENCR was identified as a shear-stress inducible lncRNA, controlling the formation of adherence-junctions at the plasma membrane level. Under static conditions, the CKAP4 protein at the plasma membrane disturbs adherence-junction formation. Under share-stress conditions, SERCA dislocates CKAP4 away from the plasma membrane, thus promoting formation of adherence-junctions and barrier stabilization [59]. Another endothelial shear-stress inducible lncRNA is LASSIE, which interacts with adherence-junction protein PECAM-1 and intermediate filament protein Nestin to promote adherence-junction association with the cytoskeleton at the luminal side of the plasma membrane [60]. Both SENCR and LASSIE were speculated to regulate trafficking of protein complexes between the ER and the plasma membrane [59,60]. This aspect, however, requires further investigation.

## 6. LncRNAs in Phase-Separated Cytoplasmic Granules

Besides organelles, separated from the cytosol by intracellular membranes, the role of liquid–liquid phase-separation in compartmentalization of cellular processes is gaining increasing attention. Phase separation is a cellular means of delimiting biochemical reactions in the intracellular milieu in liquid-like droplets, which contribute to subcellular organization [71]. Stress granules (SGs) are a well-known example of cytoplasmic phase-separated condensates, forming around RNA binding proteins and nontranslated mRNAs under conditions of limited translation initiation. Systematic investigation of the RNA content of SGs revealed that besides the expected aggregation of many mRNAs, a few lncRNAs were recruited to these structures under cell stress conditions [72,73]. Among these lncRNAs was NORAD [72,73] (Figure 1), which locates both to the cytoplasm and the nucleus and serves as a platform for PUMILIO protein assembly [74]. Knockout experiments suggest that NORAD is not essential for the formation of stress granules [72]. However, studies with artificially reconstructed phase-separated granules suggest that PUMILIO homology domain dependent recruitment of NORAD impacts on granule size and morphology [75]. Similar to SGs, P-bodies are phase-separated cytosolic structures, which are predominantly composed of proteins and mRNAs, but also contain several lncRNAs [76]. The aggregation of mRNAs in P-bodies seems to serve the negative control of translation of subsets of messengers encoding regulatory rather than house-keeper proteins [76]. Thus, different from SGs, P-bodies aggregate translationally stalled mRNAs under nonstressed conditions, as a regulatory mechanism to control protein output. Of note, under stress-conditions, the mRNomes of SGs and P-bodies are similar [77]. Different from the well-established roles of lncRNAs in nuclear condensates [78], it remains to be determined, whether lncRNAs found within P-bodies play a role in cytosolic condensate formation, composition and function. Of note, further cytosolic phase-separated RNP structures have been identified, the functions of which are less well explored. Examples are neuronal transport granules [79] and Balbiani bodies in oocytes [80]. Thus, while accumulating evidence suggests an important role of phase-separation in subcellular organization, the functions of lncRNAs in membrane-less cytoplasmic body formation are only beginning to be explored.

## 7. LncRNA Involvement in the Ubiquitin-Proteasome System

By systematically profiling the sedimentation-rates of lncRNAs on glycerol-gradients, 22 sub-groups of human phagocyte lncRNAs could be discriminated, cosedimenting with protein-components of diverse subcellular machineries. Interestingly, a prominent subgroup, comprising dozens of lncRNAs, was found to comigrate with components of the ubiquitin-proteasome system (UPS) [16]. The UPS comprises a network of ubiquitin-ligases, ubiquitinated proteins, ubiquitin-readers and the large proteasome assemblies, involved in ubiquitination-dependent protein processing and decay [81]. Ubiquitination can furthermore serve as a regulatory modification, controlling protein-activation and complex formation [81]. Recently, several cytoplasmic lncRNAs have been functionally linked to the UPS system. In innate immune cells, for instance, the signalling cascades activated in response to infectious agents are tightly controlled by ubiquitination and lncRNAs [82,83]. Detection of bacterial lipopolysaccharides by the prototypic plasma membrane spanning innate immune receptor TLR4 activates a complex signalling cascade, resulting in proinflammatory gene expression through the TLR4-MyD88 pathway and in type I interferon production through the TLR4-TRIF pathway [84]. Mirt2 is a TLR4-induced lncRNA in murine macrophages, which functions as a negative regulator of the TLR-MyD88 pathway. Mechanistically, Mirt2 binds to the TLR-MyD88 pathway component TRAF6 to block TRAF6 autoubiquitination and thus signaling pathway progression [85]. Production of antiviral and antibacterial interferons downstream of TLR4 in human cells depends on TLR4-induced lncRNA MaIL1, which promotes ubiquitination of the signalling adapter OPTN and thus progression of TLR4-TRIF signalling [16]. MaIL1 also controls the formation of cellular foci composed of ubiquitin-associated OPTN, potentially representing subcellular signalling protein platforms [16]. Similarly, murine lncRNA lnczc3h7a is upregulated upon viral infection and promotes TRIM25-dependent ubiquitination of the cytosolic antiviral innate immune-receptor RigI and thus downstream signal transduction and interferon activation [44]. Other lncRNAs regulate protein ubiquitination in the adaptive immune system to control antiviral and antitumour defense. In CD4+ T cells, for instance, LncRNA NRON was found to suppress viral replication by destabilizing viral transactivator protein Tat. Mechanistically, NRON couples the Tat protein to components of the ubiquitin-proteasome system, such as CUL4B and PSMD11, thus promoting Tat degradation [86]. In the context of hepatocellular carcinoma, upregulation of epidermal growth factor receptor (EGFR) expression in regulatory T cells (Tregs) was found to contribute to tumorigenesis [87]. An lncRNA, lnc-EGFR, was found to inhibit EGFR ubiquitination by the E3-ligase c-CBL, thereby contributing to EGFR up-regulation in T cells and Treg dependent immunosuppression [87]. Beyond the immune system, several cytoplasmic lncRNAs were found to contribute to tumorigenesis through association with ubiquitin-ligases. In glioma cells, lncRNA RP11-732M18.3 interacts with the multifunctional 14-3-3β/α proteins in the cytoplasm and recruits the ubiquitin-conjugating enzyme UBE2E1. This RNP complex promotes the ubiquitin-proteasome-dependent degradation of the cell cycle arrest inducing p21 protein, thereby contributing to cancerogenesis [88]. LncRNA BDNF-AS was reported to promote breast cancer progression by functioning as a scaffold RNA, supporting RNH1 ubiquitination by the E3 ligase TRIM21 and thus RNH1 degradation. This relieves mTOR mRNA suppression by RNH1 and promotes cell-proliferation associated mTOR signalling and thus cancer progression [89]. In non-small-cell lung cancer cells, lncRNA MetaLnc9 was found to promote cell migration and invasion. Mechanistically, MetaLnc9 interacts with and prevents from PGK1 kinase ubiquitination, thereby fostering AKT/mTOR signalling [90]. Further lncRNAs are likely involved in ubiquitination-dependent cellular processes and pathomechanisms, as indicated by the cosedimentation of dozens of lncRNAs with UPS components [16]. Of note, ubiquitinated protein substrates and ubiquitin-adapters can form aggresomes and inclusion bodies in the cytoplasm [91]. Failure to remove such aggregates by the proteasomal or autophagic pathways is involved in diseases such as amyotrophic lateral sclerosis (ALS) or frontotemporal dementia (FTD) [92]. LncRNA MaIL1 seems to be involved in the formation of ubiquitin-reader aggregates under conditions of proteasome inhibition [16]. It remains to be investigated, whether the dynamic formation and resolution of cytoplasmic ubiquitin-associated protein aggregates under homeostatic and disease conditions depend on further lncRNAs.

## 8. Further Cytoplasmic Functions of lncRNAs

Numerous other lncRNAs have been characterized, which interact with a variety of cytoplasmic components and machineries. Several lncRNAs for instance interact with cytoskeletal components. LncRNA Dreh binds to vimentin and regulates cytoskeleton structure in tumor cells [93]. LncRNA lnc-CRYBG3 was shown to interact with G-actin and inhibit actin polymerization [94]. LncRNAs SENCR and LASSIE interact with proteins interacting with the cytoskeleton at the ER and adherence-junctions [59,60] (see Section 4 and Section 5). Other lncRNAs are engaged in regulatory interactions with proteins, which do not primarily locate to larger cytoplasmic structures and machineries. Such lncRNAs regulate mRNA stability or RBP and miRNA availability in the cytoplasm by decoying or recruiting RNA-binding proteins and miRNAs, as reviewed elsewhere [12]. Taken together, besides their regulatory interactions with individual proteins or RNAs in the cytosol, accumulating evidence suggests that lncRNAs vitally participate in subcellular organization by controlling organelle functions and adopting architectural and regulatory roles at intracellular membranes, in phase-separated bodies and in large protein machineries such as the UPS or ribosomes (Figure 1).

## 9. Conclusions

In recent years, significant methodological advances have been made enabling the systematic mapping of lncRNA binding substrates and lncRNA subcellular localization patterns. Questioning the classic narrative, multiple studies suggest that lncRNAs do not primarily locate to the nucleus but also appear in the cytoplasm in large quantities [14,15,16]. Their nonrandom subcellular localization and already known implications in major cellular processes, ranging from control of cellular energy balance to translation and protein turnover (Figure 1), strongly suggest cytoplasmic lncRNAs to represent functional entities rather than by-products of transcription or RNA processing. A remaining challenge is to determine the coding potential of annotated lncRNAs exported to the cytoplasm. Reports about functional lncRNA-encoded micro-peptides demand closer investigation of the large fraction of cellular lncRNAs associating with ribosomes, to discriminate falsely annotated messengers from ribosome-regulatory lncRNAs. Among the cytoplasmic lncRNAs not bound to ribosomes, particularly many seem to be engaged in mitochondrial processes and the ubiquitin proteasome system. Little is known so far about the functions of lncRNAs found to associate with other cytoplasmic structures, such as the ER and Golgi network, vesicles or phase-separated condensates.

Preliminary work on stress-granules suggests that lncRNAs might have an architectural function during cytoplasmic phase separation. Specifically, lncRNA NORAD is enriched in stress granules and their PUMILIO-protein-dependent formation seems to depend at least partially on RNA. This is reminiscent of the requirement of architectural RNAs, such as NEAT1 or HSATIII for the assembly of phase-separated nuclear bodies [95,96]. A further indication for a role of lncRNAs in the formation of cytosolic condensates is the requirement of lncRNA MaIL1 for the UPS-dependent formation of OPTN foci [16]. OPTN serves as a ubiquitin reader, forming a poly-ubiquitin bound platform aiding the assembly of cellular signalling pathway components and promoting autophagy [43,97,98]. MaIL1 might constitute an architectural RNA involved in the formation of OPTN aggregates, helping to coordinate signalling pathway progression in the dense cytoplasmic milieu. Beyond cytosolic RNP granules, further architectural roles of lncRNAs in the cytoplasm were described. LASSIE, for instance, is required for adherence junction formation. 7SL, an ncRNA ≥ 200 nts, serves as a scaffold for SRP assembly at the ER (see Section 4). Thus, coordination of cytoplasmic protein interactions and intracellular self-organization by the aid of lncRNAs might be more widespread than previously thought.

While assembly of large protein structures such as the ribosome or proteasome results in a near-equilibrium state, the maintenance of highly dynamic structures, such as the cytoskeleton, requires constant energy input [99]. Therefore, the terms self-assembly and self-organization have been discriminated [99]. The literature available so far suggests that cytoplasmic lncRNAs participate both in self-assembly and self-organization. Scaffolding RNAs, for instance, help assembling structures such as the SRP or mitochondrial ribonuclease complexes (Section 3 and Section 4). Other lncRNAs participate in the organization of highly dynamic structures, such as the cytoskeleton and adherence junctions or cytosolic condensates formed in response to cellular stresses and environmental signals (Section 6, Section 7, and Section 8). Together with the observation that nuclear-depleted lncRNAs undergo dramatic expression changes in response to environmental cues, such as pathogen or danger signals [16,100], this suggests that lncRNAs vitally participate in the spatiotemporal coordination of cytoplasmic processes. Of note, however, the vast majority of annotated lncRNAs presently remains uncharacterized. Further investigation of the subcellular functions of these lncRNAs can be expected to confer a significantly improved understanding of the basic principles underlying the maintenance and coordination of the dense cytoplasmic milieu in health and disease.

## Figures and Tables

**Table 1:** Webtools and software to study long noncoding RNA (lncRNA) subcellular localization.

Tool	Underlying Method	URL	Ref
RNALocate	Pubmed search, community	http://www.rna-society.org/rnalocate/index.html	[26]
LncATLAS	ENCODE subcellular RNA-seq	https://lncatlas.crg.eu/	[27]
RNA-GPS	Machine learning, APEX-seq	https://github.com/wukevin/rnagps	[28]
DeepLncRNA	Machine learning, ENCODE subcellular RNA-seq	https://github.com/bgudenas/DeepLncRNA/	[29]
RNATracker	Machine learning, CeFraSeq, APEX-RIP	https://www.github.com/HarveyYan/RNATracker	[30]
LncBook	Community (Wiki)	https://bigd.big.ac.cn/lncbook/index	[31]
